# Paralysis of the Respiratory Centre from Intracranial Pressure

**Published:** 1897-06

**Authors:** J. Lacy Firth

**Affiliations:** Assistant-Surgeon to the Bristol General Hospital


### PARALYSIS OF THE RESPIRATORY CENTRE FROM INTRACRANIAL PRESSURE.[1]

BY

J. Lacy Firth, M.S., M.D. Lond., F.R.C.S. Eng.,
*Assistant-Surgeon to the Bristol General Hospital.*

My object in this paper is to draw renewed attention to a mode in which death is sometimes, if not frequently, produced, when intracranial pressure is increased; viz., by paralysis of the respiratory centre in the medulla. For this purpose I record two cases in which death was produced by such paralysis, and refer to other published examples of it, and to the work of experimental physiologists which elucidates its origin. The two cases are both of extradural hemorrhage due to head injuries.

[1] Read at the April Meeting of the Bristol Medico-Chirurgical Society.

Case 1.—The patient was a boy of ten years of age. He was admitted to the Bristol General Hospital two hours after having fallen upon his head from a first-floor window. There was not a history of immediate loss of consciousness, and on admission consciousness was complete, though the patient was somewhat drowsy. A little later he vomited. No external evidence of injury could be made out. He was kept in bed. Forty-two hours after admission respiration suddenly ceased. This occurred at ten o'clock in the morning. He had been restless all the preceding night. That was the second night after admission. He had also failed to recognise his friends during that period. Before the final cessation of breathing the Ward Sister had observed him to be breathing heavily. I saw him a minute or two later. He was then very livid, but the heart was beating regularly, producing a good radial pulse. Artificial respiration was begun at once, and for seven hours was continually kept up by relays of assistants. Throughout this period no spontaneous effort to breathe was made, but until the last two hours the heart did not flag. Towards the end the beats gradually became weaker. The respiration was stopped when the heart ceased its action. In addition to artificial respiration numerous ether injections were made, and oxygen given. The latter had the effect of dispelling a slight lividity which the artificial respiration did not completely abolish. This persistent lividity quickly increased whenever the artificial respiration was stopped a few seconds.

A necropsy was made the following day. The brain was found to be ecchymosed on the inferior surface of the frontal lobes. The left lateral lobe of the cerebellum was much flattened posteriorly. There was an extradural collection of blood in the left cerebellar fossa, extending from the foramen magnum upwards to a level half an inch above that of the groove for the lateral sinus and from the internal occipital crest outwards about two and a half inches. A fissured fracture extended downwards to the foramen magnum from a point half an inch above the groove for the left lateral sinus and half an inch to the left of the middle line. The flattening of the cerebellum was obviously produced by the displacement forwards of the dura lining the cerebellar fossa and of that forming the lateral sinus.

Case 2.—This patient was a male child, two years of age, and was admitted to the Hospital at one o'clock p.m., soon after a fall upon the head from a first-floor window. There was no scalp wound, but each parietal and temporal region was greatly swollen. The symmetrical swellings gave the head a very curious appearance. They were soft and boggy to palpation. The patient was very incompletely conscious and very pallid. He lay for the most part still and quiet, but movements of each limb were made in response to cutaneous irritation. The pupils were equal and rather wide. They acted sluggishly to light. Vomiting occurred two or three times in the first two hours after admission.

No hemorrhage from the ears. The pulse was 120; respiration normal. Two hours later the patient responded less to stimuli. The left pupil was perhaps a shade larger than the right. The right arm and leg were moved fairly briskly on stimulation, but the left hardly at all. Soon after six p.m., *i.e.* rather over five hours after admission, respiration suddenly ceased, and cyanosis became marked. Artificial respiration was begun at once, as the heart was beating regularly and yielding a fair radial pulse. I had not an opportunity of seeing the patient between three and half-past nine. At ten o'clock, *i.e.* three and a half hours after the cessation of respiration, I removed, by trephining, two discs of bone from the right side of the skull. The first disc was over the anterior branch of the middle meningeal artery. The dura here seemed very tense, and was incised, but the brain did not bulge out. I attribute this to the small size of the aperture and the viscosity of the brain. The posterior disc was divided vertically by a fissured fracture of the skull. Through the uppermost exposed part of the fissured fracture a flake or two of brain substance had exuded. No removable source of pressure was discovered through either aperture.

The heart at the end of the operation was acting as strongly as at the beginning. Artificial respiration was kept up, as well as possible, during the operation, and for two hours longer. The artificial respiration was continued until the heart's action ceased, which was six hours and ten minutes after the onset of the paralysis of the respiratory centre. During the last hours of life transient systolic cardiac bruits were frequently heard.

The reason I trephined on the right side rather than on the left, was that in my earlier observations there was a much weaker response of the left limbs to stimuli than of the right.

*Post-mortem* examination showed that if the other side had been chosen the pressure might have been much more effectually relieved. It was found that the fracture on the right side of the skull passed down and forward from near the parietal eminence to end in the right cerebellar fossa. On the left side there was a tri-radiate fissured fracture a little below and in front of the parietal eminence. Beneath this latter fracture there was an extradural collection of blood-clot. The lower part of the clot was beneath the squamous part of the temporal bone. The clot did not extend to the base of the cerebral hemisphere nor to the sphenoidal fissure in front. The left cerebral hemisphere had a saucer-like depression corresponding in area to that of the subjacent blood-clot. A ruptured branch of the posterior division of the middle meningeal artery was found, and was probably the source of the bleeding. The outer layers of the dura were torn, and the tear crossed the arterial twig mentioned. There was laceration of the cortex of the right hemisphere, the area affected being that beneath the fracture on this side. This was chiefly the angular gyrus. There was no lesion of the brain or membranes below the level of the tentorium.

It is obvious that the two cases recorded died from paralysis of the respiratory centre. This mode of death, in cases of increased intracranial pressure, is well known to many, but it has not received the general attention it deserves. There are but few recorded cases as striking as those just related. The probable reason for this is that the cases have been considered so hopeless, for the most part, that the experiment of maintaining artificial respiration for more than a few minutes has not been performed. It is very important from a practical point of view to recognise this effect of pressure on the brain, for in some cases a rapid relief of the pressure by a suitable operation will prolong life.

The following are some of the most interesting cases that have been recorded. In the *London Hospital Reports* for 1867

J. Hutchinson describes the death of a patient from meningeal hemorrhage, as follows:—"Several times his respiration entirely ceased for two or three minutes. . . . This went on for about ten minutes, when his respiration again ceased and never afterwards returned. His pulse could be felt for fully five minutes after his last respiration."

In Fagge's *Medicine* there is related a case of cerebral abscess, in which respiration suddenly ceased and the heart continued to beat for ten minutes, artificial respiration being maintained; and again, in the same book, in the chapter on cerebral tumours, occurs the following passage:—"The mode of death is often by cessation of *breathing*, the heart continuing to beat for some little time; in one instance it went on for thirty-five minutes, while artificial respiration was vigorously kept up." Mr. Horsley mentions four cases he had met with, all of them being cases of tumour of the brain. In three of these the respiration ceased suddenly as the operation of trephining was being proceeded with, and in these the operation was quickly completed and tension relieved, when the power to breathe naturally returned. Mr. Horsley writes:—"Cases of cerebral hæmorrhage, of cerebral tumour, and of depressed fracture, as well as cases of sudden and violent concussion, especially when applied in the occipital region, die from failure of respiration, and not, as is so often surmised, from failure of the heart." [1] Mr. Horsley also maintains that in those cases in which persons have been described as suddenly falling down dead, in consequence of violent blows on the head, *e.g.* from a fist or cricket ball, or from an explosion, the fatal ending has resulted from respiratory paralysis, and might in some of them have been avoided by performing artificial respiration.

Macewen[2] refers to two cases of respiratory paralysis. In one of these the heart continued to beat regularly for twenty-four hours after natural respiration had ceased. The source of pressure was a cerebellar abscess. Mr. Jalland,[3] of York, relates a case in which the breathing ceased when trephining was

[1] *Quart. M. J.*, 1894, ii. 305.
[2] *Pyogenic Infective Diseases of the Brain and Spinal Cord*, 1893, p. 321.
[3] *Lancet*, 1892, i. 527.

being proceeded with, and was not restored until pus was evacuated from a cerebral abscess. Another abscess case, where the heart continued beating for six hours, is reported from the Liverpool Royal Infirmary.[1] Drs. Sawkins and Vallack, of Sydney, give[2] notes of six cases of respiratory paralysis, all ultimately fatal. Two of their cases were of basal meningitis, with internal hydrocephalus ; the other four respectively of intra-ventricular hemorrhage, cerebral hydatids, cerebellar tumour, and malignant tumour of the base of the skull; the length of time during which the heart continued to beat varied from ten minutes to two hours.

The effects upon the functions of the brain of increasing intracranial pressure have been investigated by physiologists from early times, and the accounts given by different workers are essentially the same. Among recent workers in this field have been Horsley and Spencer, and Leonard Hill. Spencer and Horsley produced the pressure by means of a rubber bag attached to a steel tube, screwed into the skull, and externally connected to a mercurial burette, by which the rubber bag could be distended to a known degree. They introduced the bag into the fourth ventricle, in the upper part, and by moderate pressure stimulated the vagal and the vaso-motor centres, slowing the heart and raising the blood-pressure. When the bag was placed lower, upon the upper end of the cervical cord, compression produced sometimes arrest of respiration alone. They found that to arrest respiration and circulation by distension of the bag the least quantity of fluid was required when the bag was over the bulb, most when over the cerebrum, and an intermediate quantity when over the cerebellum. This is in accordance with pathological experience, for a smaller cerebellar than cerebral lesion will produce fatal compression. The culminating symptoms and fatal results of increased intracranial pressure appear to be due, not to paralysis of the vital centres by direct pressure, but to the anæmia of the bulb which is induced. Leonard Hill states that if anæmia of the mid-brain and medulla be produced experimentally, either by ligature of the carotids and vertebrals, or in any way, first spasm of the vaso-motor,

1 *Brit. M. J.*, 1893, i. 1197. 2 *Lancet*, 1895, ii. 517.

vagus, and respiratory centres is produced, causing rise of blood pressure, slow heart, and respiratory spasm; and later the same centres become paralysed, causing failure of circulation and respiration. Generally the primary cause of death is respiratory failure. "If this be compensated for by artificial respiration the heart continues to beat, and then the vaso-motor paralysis which ensues may be a secondary cause of death. . . . In some rare cases vaso-motor paralysis may be the primary cause of death." [1]

The symptoms and causes of death in cerebral compression are the same as those in cerebral anæmia, and in compression the culminating symptoms, the respiratory and circulatory failure, are produced by the arrest or diminution of the bulbar circulation, which the pressure induces. The earlier symptoms are excitatory, the later paralytic. Clinically we are familiar with this sequence. An extradural hemorrhage, for example, may first slow the heart, raise the pulse-tension, and cause deep and heavy breathing, and then secondarily paralyse respiration, lower vascular tension, and accelerate the heart's action.

Paralysis of respiration in cases of sudden concussion, or produced by bullet-wounds of the brain, which Mr. Horsley has experimentally shown to frequently occur, is perhaps not so satisfactorily explained by the theory of bulbar anæmia, for respiratory paralysis is a later symptom of such anæmia. The explosive violence, as it is termed, of the bullet rapidly moving through the viscous brain-substance, causes wide-spread injury, and damages the respiratory centre more than the other vital centres by reason of some special vulnerability of that centre, not yet capable of explanation.

To effectually relieve intracranial pressure by trephining, a considerable area of skull must usually be removed.

I am indebted to Mr. W. J. Penny and Dr. R. G. P. Lansdown for permission to publish notes of Cases 1 and 2.

---

In the discussion on this paper Dr. WATSON WILLIAMS said it would be interesting to know if there had been any marked departure from the normal pulse-rate, either in the direction of bradycardia or tachy-

[1] *The Physiology and Pathology of the Cerebral Circulation*, 1896, p. 125.

cardia.—Dr. H. Waldo had observed some cases reported of lightning stroke where recovery ensued after prolonged artificial respiration.—Dr. Theodore Fisher mentioned that if looked for, this mode of death would probably be found to occur rather frequently. He had seen the respiration gradually fail and eventually cease some ten minutes before the pulse, in a case of death from cerebral tumour.—The President drew attention to the condition in newly-born children, where, although the heart beats, yet respiration cannot be established, this being due to some injury to the respiratory centre.—Dr. Martin Randall referred to two cases of cessation of respiration of the kind Dr. Firth described: one due to hemorrhage from ruptured aneurysm of basilar artery where artificial respiration was kept up for four hours; and another of cerebral tumour in which artificial respiration was kept up, the heart beating for some hours.—Dr. Walter Swayne mentioned a *post-mortem* examination on a child delivered by version for transverse presentation in a contracted pelvis. On opening the head, in the bones of which there was a deep depression, a large clot was found underneath the dura mater. The heart continued to beat after delivery, but no efforts could induce the child to breathe.—Mr. S. H. Swayne many years ago was called to a tall, stout youth of 17, who, when at supper after rapid walking, suddenly fell forward insensible, and when Mr. Swayne came the patient had apparently ceased to breathe. Mr. Swayne used artificial respiration and venesection, with the effect of improving the heart-beat greatly, but there was no attempt at natural breathing. After more than two hours the pulsation gradually ceased. At the *post-mortem* examination a large clot of blood was found filling the ventricles of the brain.—Dr. Firth, in reply, stated that, after the onset of the paralysis, there had been no marked deviation from the normal pulse-rate.

